# Hypoxia Disrupted Serotonin Levels in the Prefrontal Cortex and Striatum, Leading to Depression-like Behavior

**DOI:** 10.3390/biology14080931

**Published:** 2025-07-24

**Authors:** Hasan Çalışkan, Koray Hamza Cihan, Seda Koçak, Gözde Karabulut, Erhan Nalçacı

**Affiliations:** 1 Department of Physiology, School of Medicine, Balıkesir University, Balıkesir 10145, Türkiye; 2 Department of Psychiatry, School of Medicine, Ankara University, Ankara 06230, Türkiye; 3 Department of Physiology, School of Medicine, Kırşehir Ahi Evran University, Kırşehir 40100, Türkiye; sdakocak@gmail.com; 4 Department of Biology, Faculty of Arts and Science, Kütahya Dumlupınar University, Kütahya 43100, Türkiye; gozdekarabulut6@gmail.com; 5 Department of Physiology, School of Medicine, Ankara University, Ankara 06230, Türkiye

**Keywords:** depression-like behaviors, forced swimming test, hypoxia, prefrontal cortex, serotonin, striatum

## Abstract

Hypoxia can adversely affect multiple organ systems. This study investigated the impact of intermittent hypoxia on serotonin levels and depression-like behaviors across distinct neuroanatomical regions. Sixteen adult female Wistar albino rats were divided into two groups: control (*n* = 8) and hypoxia (*n* = 8). The hypoxia group was exposed to a simulated altitude of 3000 for 5 h daily over 14 days. Behavioral assessments included locomotor activity (open field test) and depression-like behaviors (forced swimming test). Serotonin levels were quantified via ELISA in the prefrontal cortex, striatum, thalamus, hypothalamus, hippocampus, and serum. Intermittent hypoxia did not alter locomotor activity (*p* > 0.05) but significantly increased depression-like behavior (*p* < 0.05), accompanied by a pronounced reduction in swimming behavior (*p* < 0.0001), a marker associated with serotonergic function. Serotonin levels were significantly reduced in the prefrontal cortex (*p* < 0.005) and striatum (*p* < 0.05), while no changes were observed in other regions or serum (*p* > 0.05). These findings demonstrate that intermittent hypoxia induces depression-like behaviors and region-specific serotonin depletion, particularly in the prefrontal cortex and striatum. This underscores the need to evaluate hypoxia-related brain health implications in conditions such as sleep apnea and acute mountain sickness.

## Simple Summary

Low oxygen levels (hypoxia) can harm brain health and mood. In this study, we tested how exposing rats to hypoxia for 5 h daily over two weeks affects serotonin (a key “feel-good” chemical) and depression-like behaviors. Rats showed more depression-like behaviors. Serotonin dropped in two brain areas: the prefrontal cortex (decision-making) and striatum. This suggests that repeated, daily oxygen deprivation, such as in sleep apnea or high-altitude climbing, may disrupt the brain’s mood-regulating chemistry. Monitoring oxygen levels in at-risk conditions could help protect mental health.

## 1. Introduction

Depression is a widespread disorder worldwide and has crucial effects on individuals and society [1]. According to WHO’s Depression and Other Common Mental Disorders Report 2017, the prevalence of depression in 2015 was predicted to be 4.4% and around 322 million people [2]. Depression continues to be a serious problem in the modern world, and the burden of depression remains very high [3]. The disease has a very diverse and heterogeneous pathophysiology. Various factors play a role in the pathophysiology of depression, including neurotransmitters, neurotrophic factors, oxidative stress, inflammation, genetic predispositions, and HPA axis dysfunction [4].

Serotonin (also known as 5-hydroxytryptamine, 5-HT) is a monoamine neurotransmitter and biological amine that plays an essential role in regulating mood, digestion, sleep, and cognitive function [5,6,7]. Serotonin is a molecule that plays a key role in the pathophysiology of depression. For this reason, drugs that affect the serotonergic system are used in the current treatment of depression [8].

High-altitude exposure alters numerous physiological parameters [9]. Brain metabolism can also change remarkably in impaired oxygen conditions [10], and these conditions can affect monoamine levels in various brain areas [11]. Further, ion metabolism associated with neurotransmitter secretion can change after hypoxia [12]. Intermittent hypoxia (IH) is widely described as repeated hypoxia episodes and a subsequent normoxia period [13]. The severity of intermittent hypoxia, the duration of exposure, and the biological characteristics of the organism exposed can lead to either positive or negative outcomes [14,15,16]. Preclinical studies in the literature report both positive and negative effects of intermittent hypoxia. Studies indicate that exposure to hypoxia may increase depression-like behaviors or, conversely, produce antidepressant-like effects [15,16]. Therefore, there is no consensus on this matter. This study aims to investigate serotonin levels and depression-like behaviors in different neuroanatomical regions in adult rats subjected to intermittent hypoxia.

## 2. Materials and Methods

### 2.1. Animal

The study used 16 adult female Wistar albino rats (*Rattus norvegicus*). The subjects were selected at 10 weeks of age and weighed between 150 and 250 g. The animals were purchased from the Animal Production Laboratory at Ankara University. They were housed in the Animal Laboratory of the Department of Physiology, Faculty of Medicine, Ankara University. The rats were housed under a 12 h light/dark cycle at a constant temperature (22–25 °C) and humidity (50 ± 5%). A one-week adaptation period was implemented. No restrictions were imposed on the animals throughout the experiment. The animals had free access to food and tap water. Experiments were performed as approved by the Ankara University Experimental Animals Ethics Committee (approval reference number: 2023-9-79; meeting date: 10 May 2023). Furthermore, the experiment adhered to the international guidelines for animal studies [17].

### 2.2. Hypoxia Treatment

**Figure 1:**
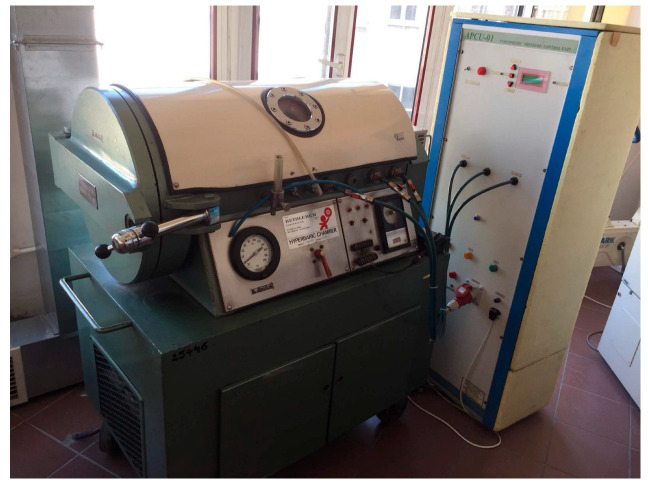
Hypoxia chamber.

Subjects were exposed to an intermittent hypoxia protocol in the hypobaric hypoxia chamber at a simulated altitude of 3000 m (partial oxygen pressure: 14%; 520 mmHg: 69.3 kPa) for 14 consecutive days, 5 h per day. The hypoxia protocol was modified according to Akat’s experiment [18]. After the intermittent hypoxia protocol, behavior tests were conducted (Figure 1).

### 2.3. Behavior Test

Behavioral testing was conducted in the Banu Ocakçıoğlu Learning and Behavior Laboratory in the Ankara University Medicine Faculty Physiology Department. Two hours before the behavioral tests began, the animals were placed in a testing room for adaptation.

### 2.4. Open Field Test

Exploratory activity in a novel environment was observed in an open-field test described previously [19]. The open-field test is used to assess locomotion and anxiety-like behaviors [20]. The open field was made up of white wood, 100 cm long, 100 cm wide, and 50 cm high, in the shape of a rectangular box and divided into 25 squares. Subjects were placed individually in the center of the open field as the starting point, and the starting point was 20 cm × 20 cm wide. The test was performed under standard lighting (110 lx) at the same time each day (08:00–12:00). Behaviors were recorded for 5 min using a ceiling-mounted digital camera. The open field was cleansed with 70% ethanol and dried with paper towels between tests.

### 2.5. Forced Swimming Test

The forced swimming test (FST) is used in the analysis of depression-like behaviors and was first described by Porsolt [21]. The FST apparatus consisted of a rectangular glass tank, 50 cm high and 25 cm in diameter. The water level was set to 40 cm, and the temperature was maintained at 25 °C. The water was renewed for each subject. Urine, feces, and fur were removed from the apparatus. Rats were subjected to a pre-test, where they were forced to swim for 15 min, and then, 24 h later, they were forced to swim for 5 min during the test. Climbing (vertical movement), swimming (horizontal movement), swimming (immobility) duration, and latency (duration of the first immobility behavior) were recorded by the camera. Behavioral analyses were conducted based on 5 min of swimming.

### 2.6. Anesthesia and Sacrifice Method

The subjects were euthanized under sodium thiopental anesthesia at a dose of 50 mg/kg. Anesthesia was administered intraperitoneally using an insulin syringe. Blood was drawn from the heart after the subjects showed no response to painful stimuli and reflexes had ceased. The organs were then removed. The brain regions of the subjects were dissected on an ice block according to the Paxinos and Watson atlas [22]. The dissected tissues were rapidly frozen with liquid nitrogen. Samples were then placed into sterile Eppendorf tubes and stored at −80 degrees Celsius.

### 2.7. Molecular Investigation

Tissues were homogenized in potassium chloride buffer at a ratio of 1:9 (0.1 g tissue: 0.9 mL, 140 mmol/L) and then centrifuged at 7000 rpm and 4 °C for 5 min. BT ELISA kit was used to measure serotonin levels in rats (Catalog no: E0866Ra, Bioassay Technology Laboratory (BT-LAB), catalog no: E0866Ra, Shanghai, China). This kit is an Enzyme-Linked Immunosorbent Assay (ELISA). The plate has been pre-coated with rat ST antibody. ST present in the sample is added and binds to antibodies coated on the wells. Then, a biotinylated rat ST antibody is added and binds to ST in the sample. Then streptavidin-HRP is added and binds to the Biotinylated ST antibody. After incubation, unbound streptavidin-HRP is washed away during a washing step. The substrate solution is then added, and color develops in proportion to the amount of rat ST. The reaction is terminated by the addition of an acidic stop solution, and absorbance is measured at 450 nm.

### 2.8. Statistical Analysis

All data were evaluated for normal distribution using the Shapiro–Wilk test. Comparisons between the two groups were performed using the Student’s *t*-test. All data were expressed as the mean ± SEM. *p* < 0.05 value was considered significant. The Resource Equation Method for animal studies suggests that two-group designs with 6–11 subjects per group are sufficient. The number n has been set to 8 due to the possibility of mortality resulting from a prolonged period of hypoxia. Additionally, the control group was evaluated within itself, and the hypoxia group was evaluated within itself in terms of serotonin levels. For this evaluation, a one-way ANOVA was performed, and the Tukey test was used as the post hoc test. Correlations were also performed using Pearson’s correlation test.

The r and *p*-values were given in the correlation analysis.

## 3. Results

### 3.1. Behavioral Results

**Figure 2:**
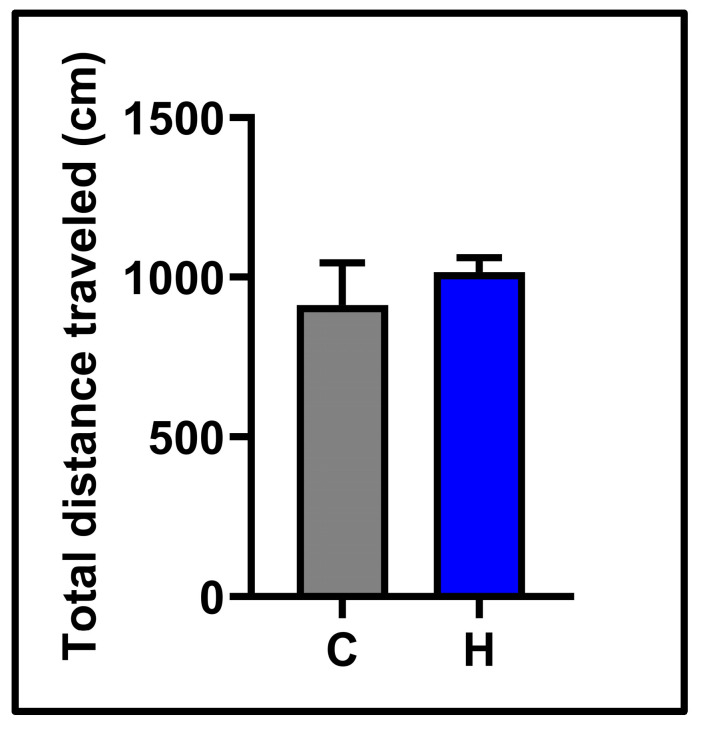
Behavioral findings in the open field test in the experimental groups: total distance traveled. C: control; H: hypoxia.

**Figure 3:**
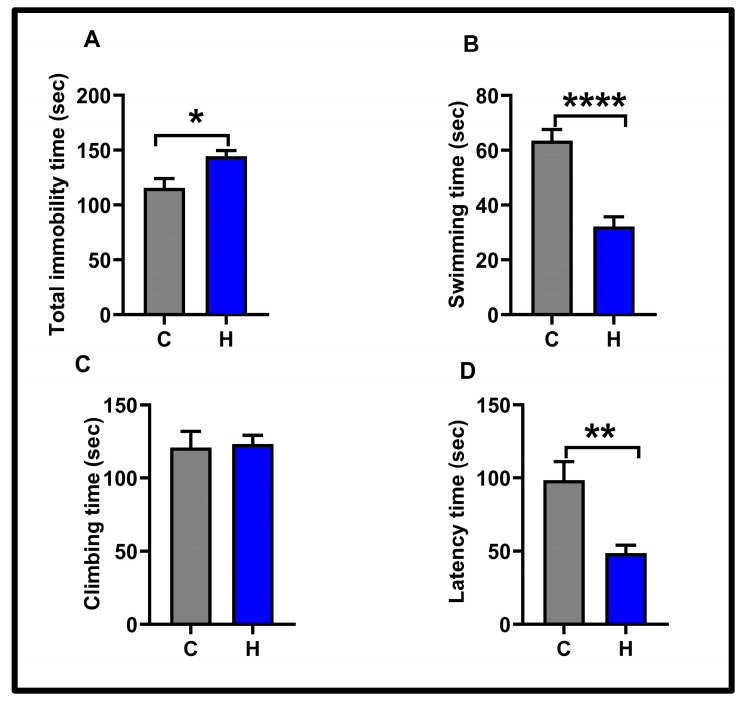
Behavioral findings in the forced swimming test in the experimental groups: (**A**) total immobility time; (**B**) swimming time; (**C**) climbing time; (**D**) latency time. Values in the graphs are presented as the means ± SEMs (* *p* < 0.05, ** *p* < 0.01, **** *p* < 0.0001). C: control; H: hypoxia; sec: second.

In the open field test, the horizontal locomotor activities of the subjects were examined. No significant difference was observed between the control (912.5 ± 131.8) and hypoxia (1015 ± 45.47) groups (See Figure 2). The results of the forced swimming test, which analyzed depression-like behavior, are presented in Figure 3. Hypoxia significantly increased the total immobility time (floating time) compared to the control group (control: 115.6 ± 8.31; hypoxia: 144.5 ± 5.09, *p* < 0.05). Swimming time was dramatically reduced in the hypoxia group (32.25 ± 3.49) vs. the control group (63.50 ± 4.06) (*p* < 0.0001). Latency time (the transition time to the first immobility) was remarkably shorter in the hypoxia group (control: 98.38 ± 12.77; hypoxia: 48.63 ± 5.34, *p* < 0.01). Climbing time was similar in the control group (120.9 ± 11) and the hypoxia group (123.3 ± 5.89, *p*> 0.05).

### 3.2. Molecular Results

**Figure 4:**
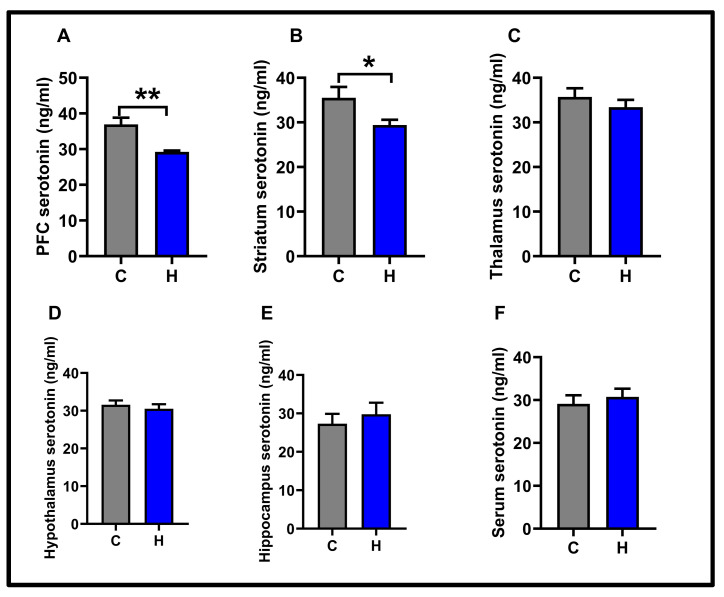
Molecular findings for the experimental groups: (**A**) PFC serotonin; (**B**) striatum serotonin; (**C**) thalamus serotonin; (**D**) hypothalamus serotonin; (**E**) hippocampus serotonin; (**F**) serum serotonin. Results are presented as mean ± SEMs (* *p* < 0.05, ** *p* < 0.01).

In this study, the levels of serotonin were measured in five different neuroanatomical regions and serum. The effect of hypoxia on serotonin levels is shown in Figure 4.

The hypoxia protocol significantly reduced serotonin levels in the prefrontal cortex (control: 36.92 ± 1.87; hypoxia: 29.22 ± 0.39, *p* < 0.01). Similarly, this protocol also caused a decrease in serotonin levels in the striatum (control: 35.53 ± 2.41; hypoxia: 29.42 ± 1.17, *p* < 0.05).

No significant differences were observed in the thalamus (control: 35.71 ± 1.93; hypoxia: 33.4 ± 1.65), hypothalamus (control: 31.58 ± 1.13; hypoxia: 30.53 ± 1.18), hippocampus (control: 27.31 ± 2.55; hypoxia: 29.80 ± 2.99), and serum (control: 29.13 ± 1.98; hypoxia: 30.75 ± 1.91) (*p* > 0.05).

**Figure 5:**
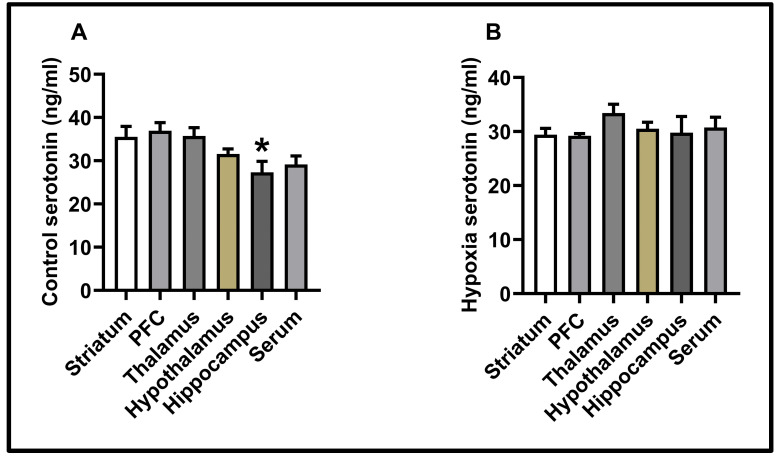
Molecular findings for the experimental groups: (**A**) control group serotonin levels; (**B**) hypoxia group serotonin levels. Results are presented as mean ± SEMs (* *p* < 0.05).

According to the results of the control group, serotonin levels in the prefrontal cortex were found to be higher than those in the hippocampus (*p* < 0.05) (see Figure 5). In contrast to the control group, there was no difference in serotonin levels within the hypoxia group (*p* < 0.05) (see Figure 4).

### 3.3. Correlation Results

**Figure 6:**
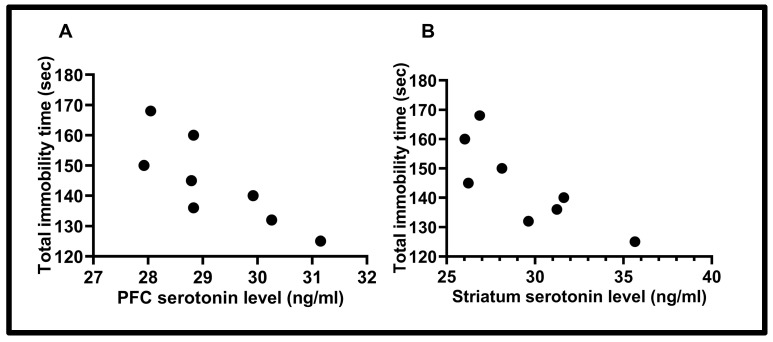
(**A**) The correlation between total immobility time (sec) and PFC serotonin level (ng/mL) (r = −0.80) (*p* < 0.05). (**B**) The correlation between total immobility time (sec) and Striatum serotonin level (ng/mL) (r = −0.81) (*p* < 0.05) (“Pearson correlation test”).

Correlations between serotonin and depression-like behavior are shown in the Figure 6. A statistically significant correlation was found between total immobility time (sec) and PFC serotonin level (ng/mL) (r = −0.80) (*p* < 0.05). Furthermore, another significant correlation was found between total immobility time (sec) and striatum serotonin level (ng/mL) (r = −0.81) (*p* < 0.05) (“Pearson correlation test”).

## 4. Discussion

The presented study is a preclinical study investigating intermittent hypoxia depression-like behavior and serotonin levels in different brain regions.

The forced swimming test is the most commonly used depression test. Although Porsolt first described FST in 1977 [21], its validity was particularly enhanced for rat studies by Detke and Lucki in 1996 [23]. With the increase in water depth in the 1990s, the behavioral patterns observed in the experiment have been broadly categorized into three categories. Floating (immobility), defined as behavioral hopelessness, occurs when rats realize that they cannot escape from the water. The subjects remain motionless on a piece of wood floating on the water. Additionally, the subjects’ horizontal swimming in the water is considered a form of swimming behavior. Swimming behavior is associated with the serotonergic system, and antidepressants that affect the serotonergic system increase swimming duration, exhibiting antidepressant-like effects [24]. Rats’ vertical swimming in water is defined as climbing and is associated with the noradrenergic system. Agents that affect the noradrenergic system also increase only this behavior [24].

In the hypoxia model we applied, immobility time, a depression-like behavior, increased significantly. Similar to other preclinical rat and mouse studies, different hypoxia applications increased immobility time [15,25]. Similarly, hypoxia applications increased depression-like behaviors in the tail suspension test, another behavioral test [26].

Our studies show that swimming behavior associated with the serotonergic system has been severely damaged and reduced. No change was observed in climbing behavior. Hypoxia studies in the literature focus on the decrease or increase in depressive behavior. Our study shows an increase in depression-like behavior and explains that this increase is associated with a decrease in serotonergic swimming behavior serotonin is one of the most important neurotransmitters in mood modulation [27]. Decreases in serotonin levels in the central nervous system and receptor desensitization cause depression [28]. The present study investigated cortical and subcortical structures. A decrease in serotonin levels was observed in the prefrontal cortex and striatum. No significant changes were observed in the thalamus, hypothalamus, hippocampus, and serum. A correlation was found between the decrease in serotonin levels in both the prefrontal cortex and striatum and the increase in depressive behavior in the forced swimming test.

Serotonin-producing neurons are susceptible to low oxygen levels. Tryptophan hydroxylase (TPH) is the rate-limiting enzyme in 5-HT synthesis. TPH is oxygen-dependent, and sufficient oxygen levels must be maintained to preserve its enzymatic activity. Therefore, serotonergic neuronal oxygen levels are susceptible to changes in oxygen levels [29,30].

Activation of the enzyme indoleamine 2,3-dioxygenase (IDO) causes tryptophan to be directed towards the kynurenine pathway instead of serotonin [31]. Song et al. reported that hypoxia induces IDO production in dendritic cells [32]. IDO, which converts tryptophan to kynurenine, is essential in the pathophysiology of depression [33]. Kynurenine metabolites, including 3-hydroxy-kynurenine and quinolinic acid display toxic effects on neurons. These poisonous substances can cause oxidative stress, apoptosis, and atrophy [34]. Hypoxic conditions can lead to the release of proinflammatory cytokines from both neurons and glial cells [35]. This can lead to brain toxicity [36]. Numerous studies have demonstrated that serotonin production decreases as a result of inflammation [36,37]. Both the oxygen sensitivity of the speed-limiting enzyme and the triggering of IDO production by hypoxia may contribute to the serotonin depletion observed in our study.

The interaction between the prefrontal cortex and the striatum has a potent effect on cognitive processes [38]. The information derived from the prefrontal cortex is processed in the striatum; thus, in the striatum, information is funneled [39]. The PFC and striatum are critical neuroanatomical connections associated with depression [40,41]. In our previous neuroinflammation model, we found that serotonin levels in the prefrontal cortex and striatum were significantly reduced [37]. It has been reported that BDNF is transported between the PFC and striatum via an anterograde transport pathway [42]. Brain-derived neurotrophic factor (BDNF) levels in these two regions influence each other. In two different experimental designs that we previously conducted, BDNF levels in the PFC-striatum were found to be parallel [37,43]. The transport of serotonin metabolites between the PFC and striatum may also occur similarly. Sleep apnea and the high-altitude cerebral edema model are being investigated in preclinical studies using hypoxia [44,45]. Depression incidence is high in these diseases [46,47]. Serotonin production impairment may contribute to the process in these pathophysiologies where hypoxia is present.

Kanekar et al. demonstrated that chronic exposure to altitudes of 4500–10,000 feet causes a decrease in serotonin levels in the prefrontal cortex and striatum, and an increase in behavioral despair in female rats [48]. These results support our investigation. However, unlike this model, intermittent hypoxia was used in our study. Chronic intermittent hypoxia, as seen in obstructive sleep apnea syndrome, leads to neural damage, depression, and fatigue [49]. Our model more realistically mimics the episodic nocturnal hypoxia observed in sleep-related respiratory disorders such as obstructive sleep apnea syndrome. It is thought that changes in oxygen levels may affect brain monoamine systems differently than continuous hypoxia. Specifically, our study establishes a direct and strong correlation between serotonin depletion in the prefrontal cortex and striatum and depression-like floating behavior in the forced swimming test. Our findings provide a clinically meaningful model for serotonergic dysfunction and behavioral despair caused by intermittent hypoxia, shedding light on the links between sleep-related hypoxia and mood disorders.

Preclinical studies also suggest that mild hypoxia may be beneficial for mental health [50,51,52]. Mild repeated episodes of hypoxia have long been used for training pilots, mountaineers, and athletes [53]. However, hypoxia can be detrimental, depending on the protocol type, duration, altitude, and quantity of partial oxygen [54,55,56].

The presented study has certain limitations. From a translational medicine perspective, experiments should also be conducted with male subjects to fully reflect public health. Due to budget and time constraints, this was not possible in the present study. The investigation of other metabolites involved in the pathophysiology of depression may also be helpful in future studies. In particular, assessing levels of BDNF, other neurotrophic factors, such as artemin, persephin, and neuturin, as well as the nerve growth factor family, other neurotransmitters like norepinephrine and dopamine, or kynurenine metabolites, could provide a deeper mechanistic understanding.

## 5. Conclusions

Our study demonstrates that intermittent hypoxia induces region-specific serotonin depletion, with the prefrontal cortex and striatum showing significantly reduced serotonin levels compared to other examined brain regions. These neurochemical changes were behaviorally correlated with increased depression-like manifestations, as evidenced by increased immobility time in the forced swimming test. Notably, we observed a strong negative correlation between serotonin reduction in the prefrontal cortex/striatum and the expression of depressive behaviors, suggesting a potential causal relationship. The differential vulnerability of brain regions to hypoxic insult, with prefrontal and striatal areas being particularly sensitive, may have important implications for understanding mood disorders in hypoxia-associated conditions such as sleep apnea and high-altitude exposure.

## Data Availability

The data that support the findings of this study are available from the corresponding author upon reasonable request.

## Abbreviations

The following abbreviations are used in this manuscript:

BDNF	Brain-derived neurotrophic factor
FST	Forced swimming test
IDO	Indoleamine 2,3-dioxygenase
PFC	Prefrontal cortex
LD	Linear dichroism
